# Correction: Razzaq et al. Omics and CRISPR-Cas9 Approaches for Molecular Insight, Functional Gene Analysis, and Stress Tolerance Development in Crops. *Int. J. Mol. Sci.* 2021, *22*, 1292

**DOI:** 10.3390/ijms26115031

**Published:** 2025-05-23

**Authors:** Muhammad Khuram Razzaq, Muqadas Aleem, Shahid Mansoor, Mueen Alam Khan, Saeed Rauf, Shahid Iqbal, Kadambot H. M. Siddique

**Affiliations:** 1 Soybean Research Institute, National Center for Soybean Improvement, Nanjing Agricultural University, Nanjing 210095, China; khuram.uos@gmail.com (M.K.R.); muqadasaleem@gmail.com (M.A.); 2 National Institute for Biotechnology and Genetic Engineering, Faisalabad 38000, Pakistan; 3 Department of Plant Breeding and Genetics, Faculty of Agriculture and Environment, The Islamia University of Bahawalpur, Punjab 63100, Pakistan; mueen.alam@iub.edu.pk; 4 Department of Plant Breeding and Genetics, College of Agriculture, University of Sargodha, Sargodha 40100, Pakistan; saeedbreeder@hotmail.com; 5 Laboratory of Fruit Tree Biotechnology, College of Horticulture, Nanjing Agricultural University, Nanjing 210095, China; 2017204045@njau.edu.cn; 6 The UWA Institute of Agriculture, The University of Western Australia, Perth, WA 6001, Australia

In the original publication [1], reference [61] Vickers, N.J. Animal communication: When I’m calling you, will you answer too? *Curr. Biol.* **2017**, *27*, R713 was mistakenly added. The correct reference is [61] Arbona, V.; Manzi, M.; de Ollas, C.; Gómez-Cadenas, A. Metabolomics as a tool to investigate abiotic stress tolerance in plants. *Int. J. Mol. Sci.* **2013**, *14*, 4885. With this correction, the reference citation order is kept the same as before. The authors state that the scientific conclusions are unaffected. This correction was approved by the Academic Editor. The original publication has also been updated.

## References

[1] Razzaq M.K., Aleem M., Mansoor S., Khan M.A., Rauf S., Iqbal S., Siddique K.H.M. (2021). Omics and CRISPR-Cas9 Approaches for Molecular Insight, Functional Gene Analysis, and Stress Tolerance Development in Crops. Int. J. Mol. Sci..

